# A process mining approach for clinical guidelines compliance: real-world application in rectal cancer

**DOI:** 10.3389/fonc.2023.1090076

**Published:** 2023-05-17

**Authors:** Mariachiara Savino, Giuditta Chiloiro, Carlotta Masciocchi, Nikola Dino Capocchiano, Jacopo Lenkowicz, Benedetta Gottardelli, Maria Antonietta Gambacorta, Vincenzo Valentini, Andrea Damiani

**Affiliations:** ^1^ Diagnostica per immagini, Radioterapia Oncologica ed Ematologia, Università Cattolica del Sacro Cuore, Rome, Italy; ^2^ Diagnostica per Immagini, Radioterapia Oncologica ed Ematologia, Fondazione Policlinico Universitario Agostino Gemelli IRCCS, Rome, Italy; ^3^ Real World Data Facility, Gemelli Generator, Fondazione Policlinico Universitario Agostino Gemelli IRCCS, Rome, Italy

**Keywords:** process mining, conformance checking, process discovery, clinical guidelines, computer-interpretable clinical guidelines, rectal cancer, treatment pathways, evidence - based medicine

## Abstract

In the era of evidence-based medicine, several clinical guidelines were developed, supporting cancer management from diagnosis to treatment and aiming to optimize patient care and hospital resources. Nevertheless, individual patient characteristics and organizational factors may lead to deviations from these standard recommendations during clinical practice. In this context, process mining in healthcare constitutes a valid tool to evaluate conformance of real treatment pathways, extracted from hospital data warehouses as event log, to standard clinical guidelines, translated into computer-interpretable formats. In this study we translate the European Society of Medical Oncology guidelines for rectal cancer treatment into a computer-interpretable format using Pseudo-Workflow formalism (PWF), a language already employed in pMineR software library for Process Mining in Healthcare. We investigate the adherence of a real-world cohort of rectal cancer patients treated at Fondazione Policlinico Universitario A. Gemelli IRCCS, data associated with cancer diagnosis and treatment are extracted from hospital databases in 453 patients diagnosed with rectal cancer. PWF enables the easy implementation of guidelines in a computer-interpretable format and visualizations that can improve understandability and interpretability of physicians. Results of the conformance checking analysis on our cohort identify a subgroup of patients receiving a long course treatment that deviates from guidelines due to a moderate increase in radiotherapy dose and an addition of oxaliplatin during chemotherapy treatment. This study demonstrates the importance of PWF to evaluate clinical guidelines adherence and to identify reasons of deviations during a treatment process in a real-world and multidisciplinary setting.

## 1 Introduction

From 1990’s, the management of rectal cancer has resulted in progressively improved outcomes through the integration of new treatment options, substantially improving 5-year cancer survival rates (1, 2).

According to a report by the Surveillance Epidemiology and End Results (SEER), from 1998 to 2007, 67.8% of patients with stage II and III rectal cancer were treated with neoadjuvant intent, with a significant increase from 17% in 1998 to 51% in 2007 (3). The latest data from the National Center for Health Statistics report that colon-rectal cancer (CRC) mortality rates decreased by 3% per year in individuals aged 65 years from 2008 to 2017 (4). Starting from this, in the following years to the present, the role of surgical, radiotherapy and chemotherapy treatments has evolved in the perspective of personalised medicine. Indeed, depending on patient selection, different approaches of treatment intensification, both chemotherapy and radiotherapy, have been explored, along with the evolution of non-operative treatment approaches aiming at organ-preservation (5). With the introduction of Evidence-based Medicine, that is, information-driven and evidence-based clinical management (6), several national and international societies published recommendations for clinical practice on treatment choices for rectal cancer. The publication and dissemination of these documents aimed to achieve standardized management of rectal cancer in both early and advanced stages. The latest guidelines of the European Society of Medical Oncology (ESMO), published in 2017 (7), group patients according to the clinical presentation of the disease into early, intermediate, locally advanced, and advanced rectal cancer and suggest treatment options tailored to each subgroup. However, despite the clinical guidelines (CGs) aiming to improve the quality of care and reduce healthcare costs, the literature review shows inconsistent full adherence to guidelines, including those for rectal cancer (3, 8). Almost one third of patients with stage II/III rectal cancer receive no pelvic radiotherapy and social, racial, gender and age disparities were reported (3). Moreover, potential reasons for this can be found in the CGs’ aim to standardize clinical processes by focusing on subgroups of patients, often only taking into account information on the stage of the disease, whereas, during clinical practice, physicians may have to adapt general guidelines to the characteristics of the individual patient (9). Consequently, the final treatment decision is usually influenced by additional medical knowledge, not reported in clinical guidelines, that may lead to deviations from them.

In recent years, process mining in healthcare has emerged as a promising field that exploits the large amount of real-world data routinely acquired in EHRs (Electronic Health Records) to improve hospital processes (10).

Process mining is based on three conceptual elements: process discovery, conformance checking, and process enhancement. Process discovery automatically creates a process model that describes the behaviors observed in a real process, conformance checking compares a real-world process with a predefined process model, while process enhancement returns a process model that fits better a given real-world process (11). Conformance checking therefore offers the opportunity to investigate the extent to which CGs are used in clinical practice, providing a useful set of tools to assess the adherence of actual hospital processes, represented in the form of event logs stored in the hospital’s data warehouse, to a standard model process (11, 12). The starting point of CGs analysis is the formalization of text-based clinical guidelines published in journals as computer-interpretable guidelines (CIGs) (13). Over the past 20 years, CIGs have proposed many solutions, such as GLIF (14), PROForma (15), Asbru (16), EON (17), BPMN (18) and time boxing formalism (19). The main purpose of CIGs has been to integrate clinical guidelines into the careflow and to build decision support systems based on CIGs to provide patient-specific recommendations (20, 21). One of the main barriers to the implementation of these decision support systems was the integration of CIGs with EHR, but nowadays process mining has provided fertile ground for new applications of CIG, allowing direct communication with hospital information systems (22, 23). In the oncology field, several authors have assessed compliance with standard CGs by integrating conformance checking techniques with CIGs. For example, to investigate surveillance process of melanoma patients, clinical guidelines were represented with BPMN (Business Process Modeling Notation) and time boxing (19, 24). Furthermore, BPMN was used to check conformity to guideline-based therapy recommendations in colorectal cancer (25).

However, CIGs representations are not always easily interpreted by clinicians, especially if they have been developed to support corporate organizations, such as BPMN (26), and their implementation could be not straightforward. Among the set of possible formalisms designed to represent CGs, the Pseudo-Workflow formalism (PWF) (27), already used in oncology domain to represent CGs (28, 29), has been implemented within pMineR (30, 31), a software library specifically designed for process mining in healthcare.

In this study we analyze conformity to international ESMO guidelines in a real-world cohort of rectal cancer patients; the PWF was used as an easily implementable and interpretable formalism to produce CIGs.

## 2 Materials and methods

### 2.1 Data

All adult patients with histological diagnosis of non-metastatic rectal cancer treated at the Fondazione Policlinico Universitario A. Gemelli IRCCS from January 2017 to December 2021 were included in the study. Patients without a surgical or chemoradiation treatment and patients with missing clinical staging information were excluded from the final dataset.

According to ESMO guidelines, patients were grouped into four risk categories based on initial clinical presentation: early, intermediate, locally advanced and advanced.

Data on clinical staging, radiotherapy, chemotherapy, surgery and follow up were automatically collected from a database in the radiotherapy department routinely managed by data managers and validated by dedicated physicians. The database supports data collection for the radiotherapy department and provides an underlying ontology structure, i.e., a disease-specific terminological system for standardized data collection (32). Clinical data were automatically extracted from the database using the hospital’s data science facility Gemelli Generator Real World Data (G2 RWD) (33). This facility oversees the design and execution of research studies in the hospital’s departments and provide a repeatable framework covering all the main steps of a data science and research project, from defining the ontology of the specific study to data analytics and AI model development.

The clinical data were shaped in the form of event log including five different types of events related to the cancer diagnosis and treatment:

- Clinical staging. TNM staging assessment based on examinations performed prior to surgery and treatment.
- Neoadjuvant radiotherapy. Preoperative radiotherapy treatment.
- Neoadjuvant chemotherapy. Preoperative concomitant chemotherapy treatment.
- Surgery. Surgical procedures performed for rectal cancer. We included all types of surgery performed in our population in the event log. The ESMO guidelines prescribe two main types of surgery: TEM (transanal endoscopic microsurgery) and TME (total mesorectal excision).
- Watch and wait. An organ-preserving approach in which surgery is not performed after chemoradiotherapy (CRT) and the patient’s condition is carefully monitored with regular follow-ups. This approach is used in complex patients or those who have achieved a complete clinical response after neoadjuvant treatment.

To check compliance with the ESMO guidelines recommendations, timestamps and a set of attributes associated with each type of event were also collected and inserted as columns in the event log (**Table 1**).

**Table 1:** Event log characteristics: event types, event names, event attributes and number of missing values for each attribute.

Event type	Event name	Event Attributes	Number of missing values
Clinical staging	Staging_C	TNM risk category (early, intermediate, locally advanced, advanced)	0
Neoadjuvant radiotherapy	Nad_rt	Total dose delivered	5
		Fraction dose delivered	6
Neoadjuvant chemotherapy	Nad_ct	Chemotherapy agents	4
Surgery	Surgery	Type of local surgery	3
Watch and wait	Watch_wait	-	

### 2.2 ESMO clinical guidelines

The ESMO Clinical Practice Guidelines are intended to provide the user with a set of recommendations for the best standards of cancer care, based on the findings of evidence-based medicine in accordance with the ESMO Standard Operating Procedures for the development of Clinical Practice Guidelines.

For rectal cancer, the ESMO Clinical Practice Guidelines provide recommendations for diagnosis, treatment and follow-up. The latest European Society of Medical Oncology ESMO guidelines, published in 2017, proposed a classification according to the clinical presentation of the disease into early, intermediate, locally advanced and advanced rectal cancer.

**Figure 1:**
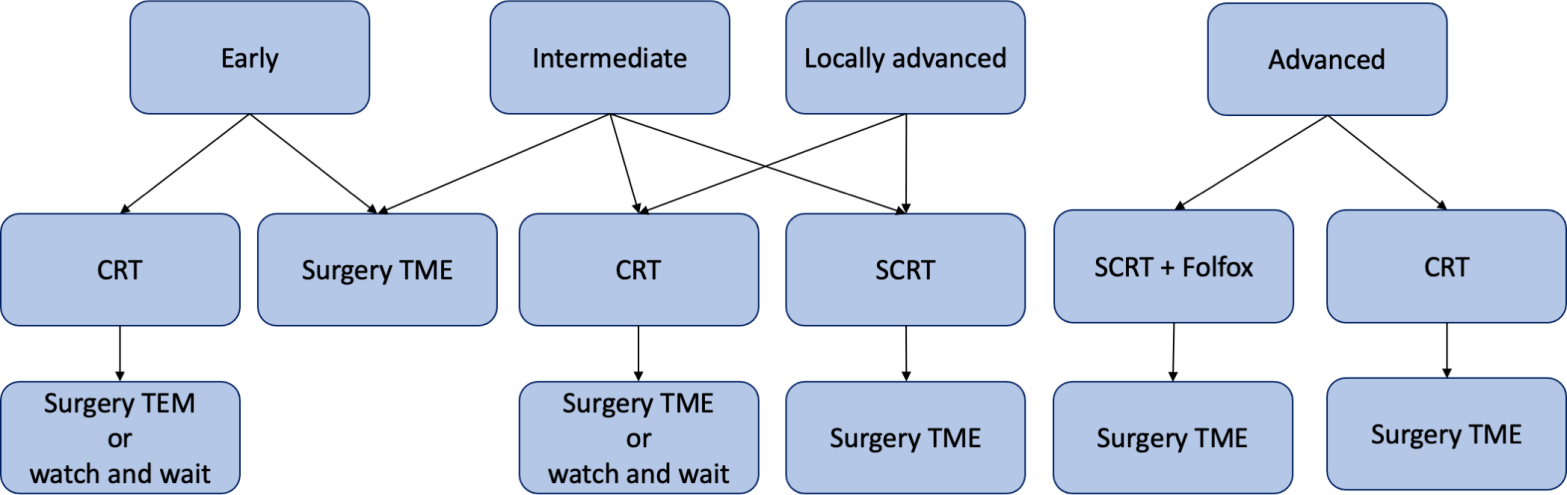
Graphical representation of ESMO recommendations for rectal cancer treatment in early, intermediate, locally advanced and advanced risk categories. CRT, chemoradiotherapy; SCRT, short-course radiotherapy; TME, total mesorectal excision; TEM, transanal endoscopic microsurgery; Folfox, leucovorin/fluorouracil/oxaliplatin.

Specific treatment options are recommended for each category (**Figure 1**):

1. Neoadjuvant chemoradiotherapy (nCRT) followed by TEM surgery or watch and wait (W&W) approach (‘Early’ risk category).
2. TME surgery (‘Early’ and ‘Intermediate’ risk categories).
3. nCRT followed by TME surgery or W&W approach (‘Intermediate’, ‘Locally Advanced’ and ‘Advanced’ risk categories).
4. Short-course radiotherapy (SCRT) followed by TME surgery (‘Locally Advanced’ risk category).
5. SCRT followed by Folfox chemotherapy and TME surgery (‘Advanced’ risk category).

When nCRT (also known as long course radiotherapy) is recommended, the guidelines prescribe radiotherapy with a delivered dose in the range of 45- 54 Gray and a fraction delivered dose in the range of 1.8- 2.0 Gray, followed by chemotherapy with 5-fluorouracil or capecitabine. SCRT, on the other hand, requires a delivered dose of 25 Gray. Only for patients in the advanced risk group, SCRT is recommended in combination with Folfox, a chemotherapy regimen consisting of leucovorin, fluorouracil and oxaliplatin.

In the present work we have focused on neoadjuvant treatment and surgery, excluding adjuvant therapies, as the level of scientific evidence for sufficient relative benefit is much lower than for colon cancer.

### 2.3 Conformance checking

Among the various process mining software available (e.g., ProM, Disco,…), we used pMineR^1^ (31), an R-based software, specifically designed for process mining applications in the healthcare domain, to assess the conformity of our real-world event log to the ESMO guidelines. The pMineR software was developed to support physicians in analyzing the actual processes performed within the hospital and has two main features that make it particularly suitable for healthcare applications: (1) Process mining analyses are performed within R, which is one of the most widely used statistical frameworks in the medical field. (2) It provides graphical representations of processes that are easily interpreted by clinicians.

pMineR performs conformance checking by working with an internal formalism called “Pseudo-Workflow” (PWF). A specific engine implemented in the pMineR software interprets and executes clinical guidelines written in PWF.

**Figure 2:**
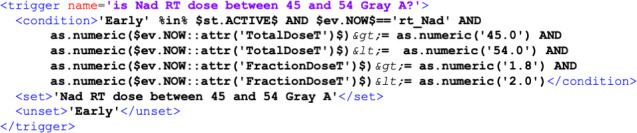
Example of rule, which is called trigger in PWF. Each rule is identified with a name (“is Nad RT dose between 45 and 54 Gray A?”) and includes a condition (e.g., if the patient belongs to the early risk group and the event is neoadjuvant radiotherapy with a total dose between 45 and 54 Gray and a fraction dose between 1.8 and 2.0). If the condition is met the patient transitions from one state (“Early”) to another (“Nad RT dose between 45 and 54 Gray A”).

PWF describes CGs in terms of two basic components: nodes (or states) and rules (called triggers in PWF). Each rule contains a condition and some effects: for each rule, a condition is tested, and if the condition is true, an effect is produced that changes the state of the patients. **Figure 2** shows an example of the rule for the ESMO guidelines, it describes the transition from the initial early risk group to the neoadjuvant radiotherapy treatment. The rule is called “is Nad RT dose between 45 and 54 Gray A?”. It states that, if the patient is in the early risk group and receives neoadjuvant radiotherapy treatment with a delivered dose within the specified range (45 – 54 Gray for total delivered dose and 1.8 – 2.0 Gray for fraction delivered dose), the patient status changes from ‘Early’ to ‘Nad RT dose between 45 and 54 Gray’.

Given a trace in the event log, the PWF engine reads all the events in chronological order and when the condition is met the patient status is updated.

The PWF formalism works directly on data in the form of an event log and conditions can work on events and attributes of the event log, i.e. in the previous example the condition works on the current event (rt_Nad) and the corresponding attributes (TotalDoseT and FractionDoseT).

The same rule structure is used to represent the entire workflow of the ESMO guidelines, resulting in a final XML file that can be interpreted by an engine in the pMineR software to generate a workflow diagram.

To assess conformity to the CGs, the real event log is run against the PWF version of the CGs, an engine reads the list of events and tests whether one or more ‘‘rules” can be fired for each event.

The output of the software is a workflow diagram that represents the CG and shows the number of patients that have activated each transition. The software calculation also produces an XML file that can be queried to identify adherent and nonadherent patients at each guideline node for further analysis, e.g. to investigate the impact of CGs adherence on important clinical outcomes or to discover and analyse the different treatment pathways carried out by non-compliant patients.

### 2.4 Process discovery

In the set of process discovery algorithms provided by pMineR, the CareFlow miner algorithm (34) combines methods derived from sequential pattern mining and temporal data mining to automatically detect the most frequent pathways in a real event log. To find the most frequent sequence of events, CareFlow miner algorithm computes the support for each of them. The support of a sequence of events is defined as the number of patients who experience it divided by the total number of patients.

Key features of the CareFlow miner are its ability to overcome the “spaghetti” effect, a common problem in process discovery in healthcare applications due to the heterogeneity of patient pathways, and to provide an easily interpretable visualization in the form of a directed acyclic graph (DAG).

To eliminate the spaghetti effect, a threshold is applied on the supports and only the most frequent transitions, involving a percentage of patients greater than the threshold, are displayed.

The aim of this discovery process was to provide a general overview of the real process and an easy-to-use representation to analyse the paths of the patients who did not match the CGs.

## 3 Results

### 3.1 Event log

**Figure 3:**
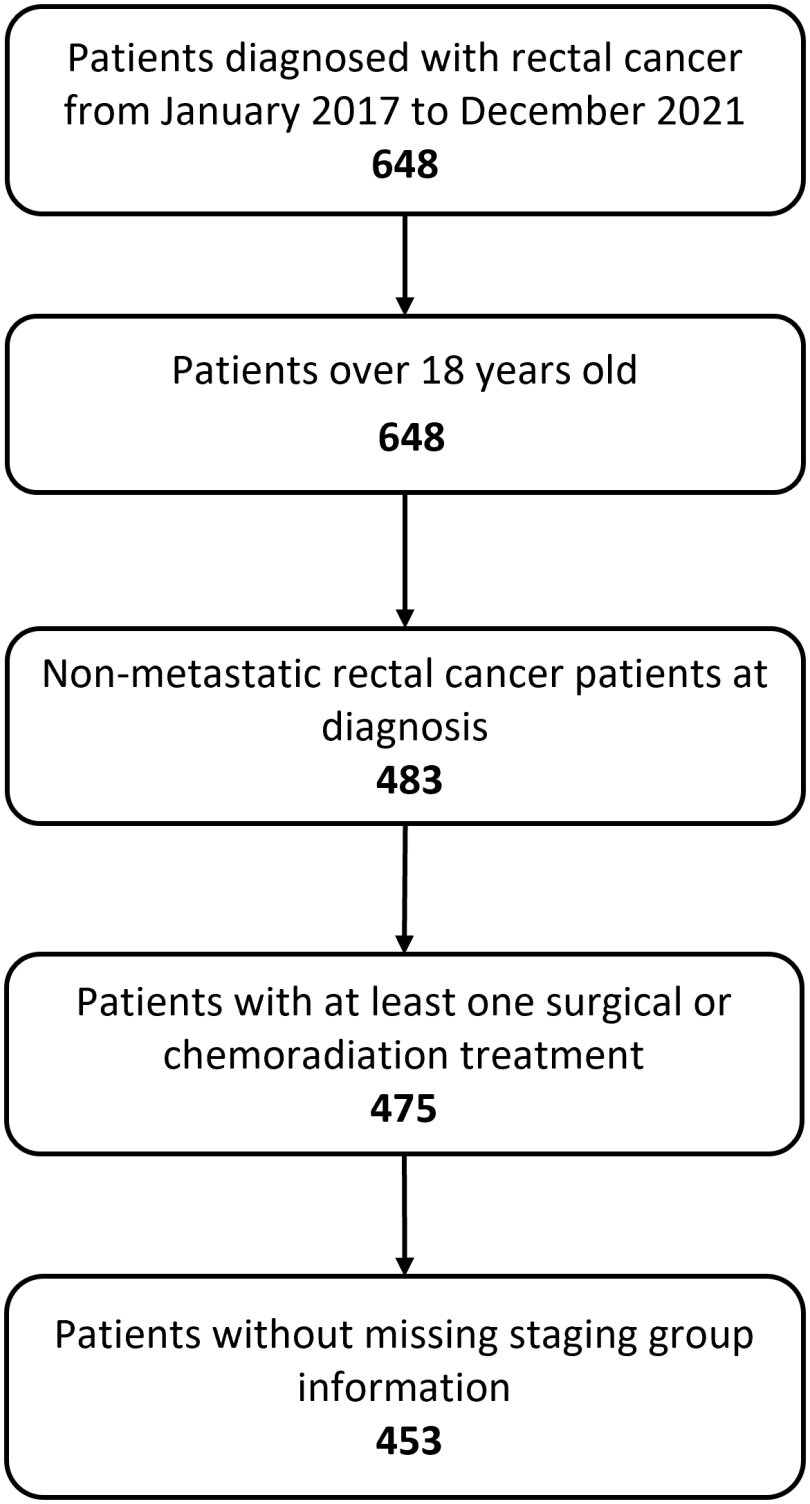
Flow chart showing study inclusion and exclusion criteria. 483 adult patients were diagnosed with non-metastatic rectal cancer. We excluded 30 patients who either did not undergo treatment or had missing information regarding staging. The final dataset includes 453 patients.

The final event log included 1895 rows, each row representing a distinct event associated to 453 non-metastatic rectal cancer patients treated in Fondazione Policlinico Gemelli IRCCS from January 2017 to December 2021 (**Figure 3**).

**Table 1** displays number of missing values for each event attribute. Given small number of missing values for almost all attributes in our event log, we decided to keep them and to use the whole dataset for analysis.

The median age value of our population was 66 years (IQR = 17) and the number of females was 192 (42.38%).

**Figure 4:**
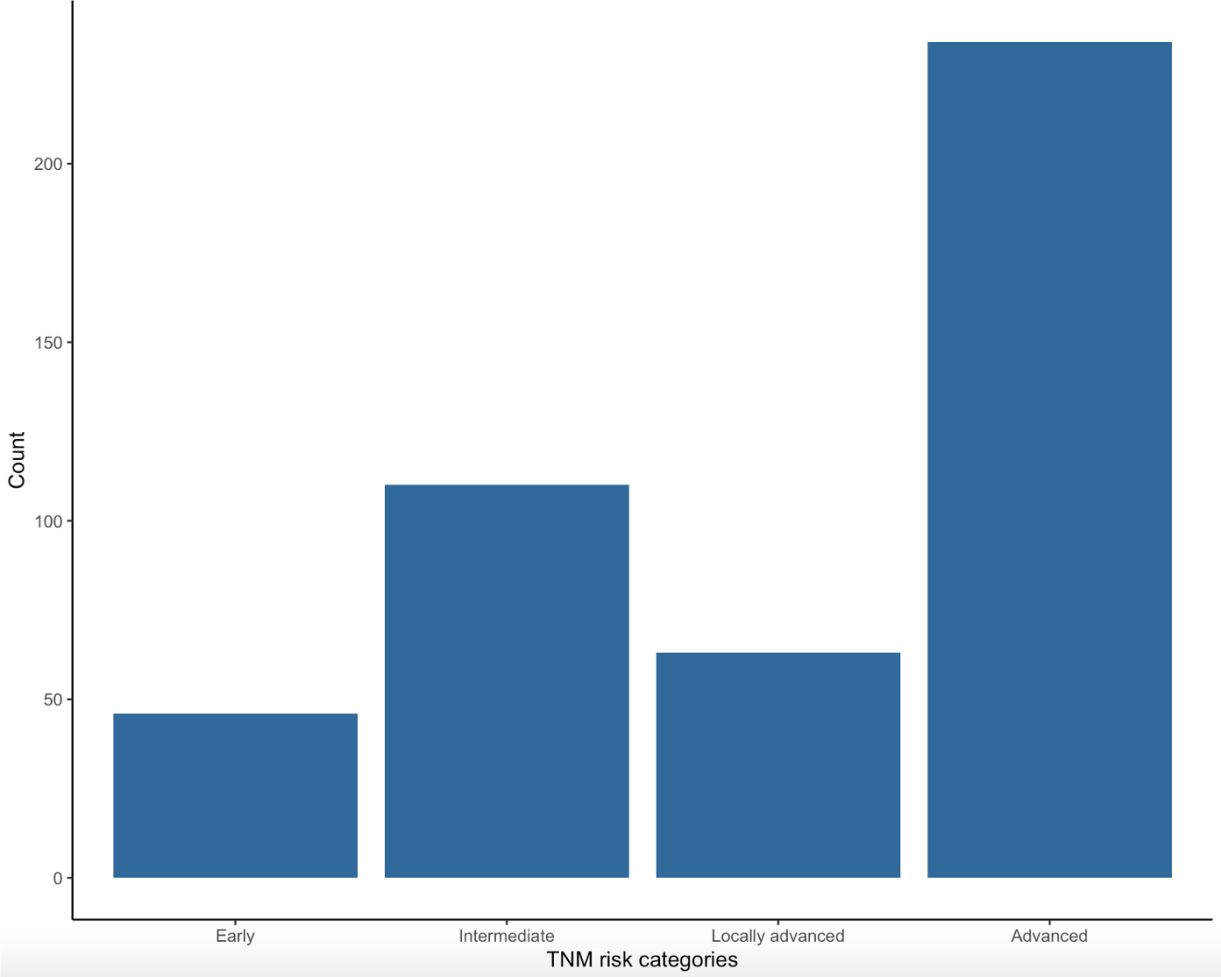
Distribution of patients in the four TNM risk categories at the moment of diagnosis: early, intermediate, locally advanced and advanced.

**Figure 4** shows distribution of patients in the four TNM risk categories on the basis of initial clinical presentation. The highest percentage of patients were in the advanced (234, 51.66%) category followed by intermediate (110, 24.28%), locally advanced (63, 13.91%) and early (46, 10.15%) categories.

### 3.2 Pseudo-workflow version of ESMO guidelines

**Figure 5:**
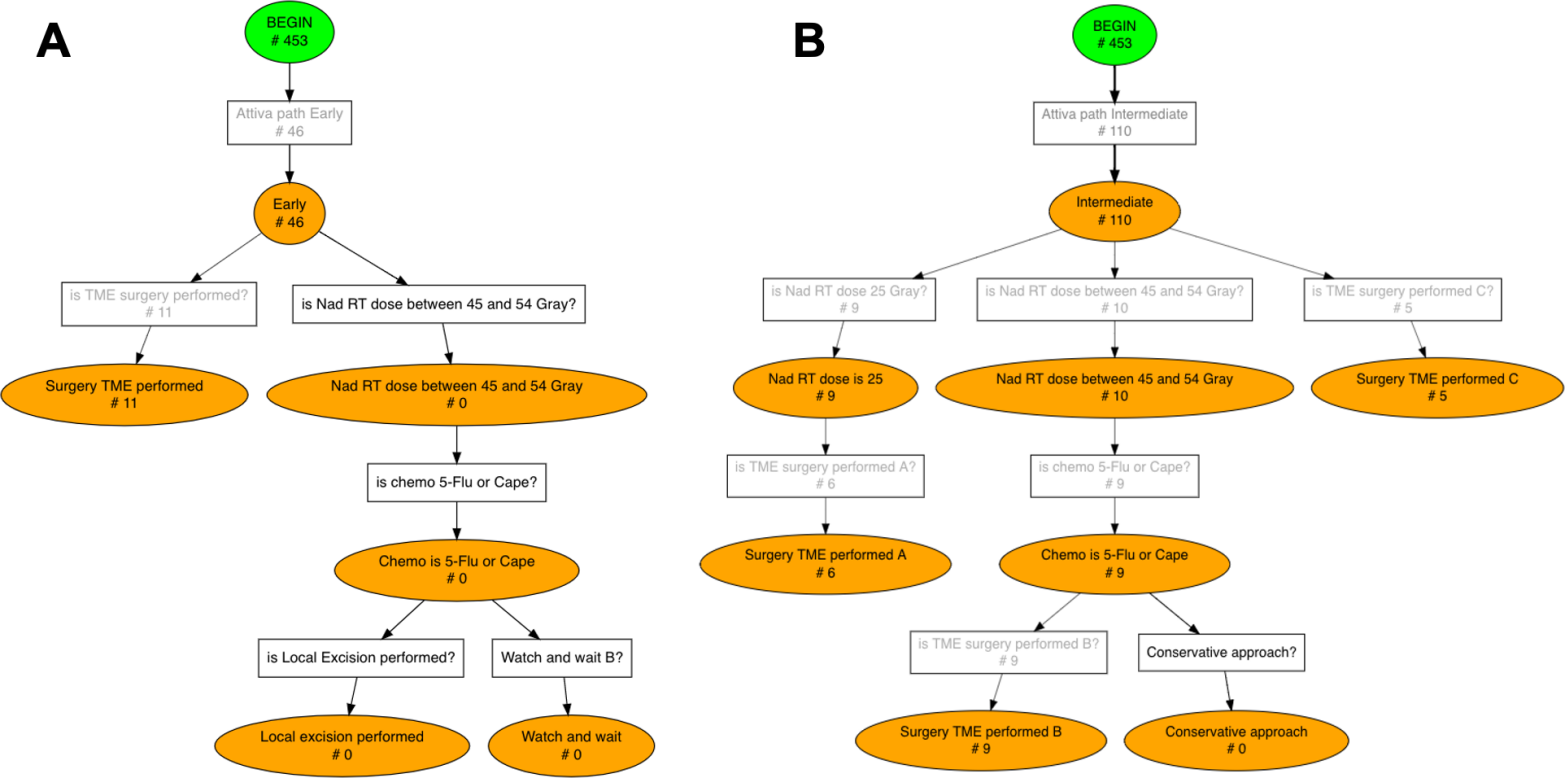
Conformance checking results for Early **(A)** and Intermediate **(B)** risk groups. The number of patients who activated each rule, i.e. each transition, is displayed on the Pseudo-Workflow diagrams representing ESMO guidelines’ recommendations: boxes are rules, circles are statuses. Only patients that reach final nodes are patients fully compliant with ESMO guidelines, all others deviate from the guidelines at some point in their treatment pathway.

**Figure 6:**
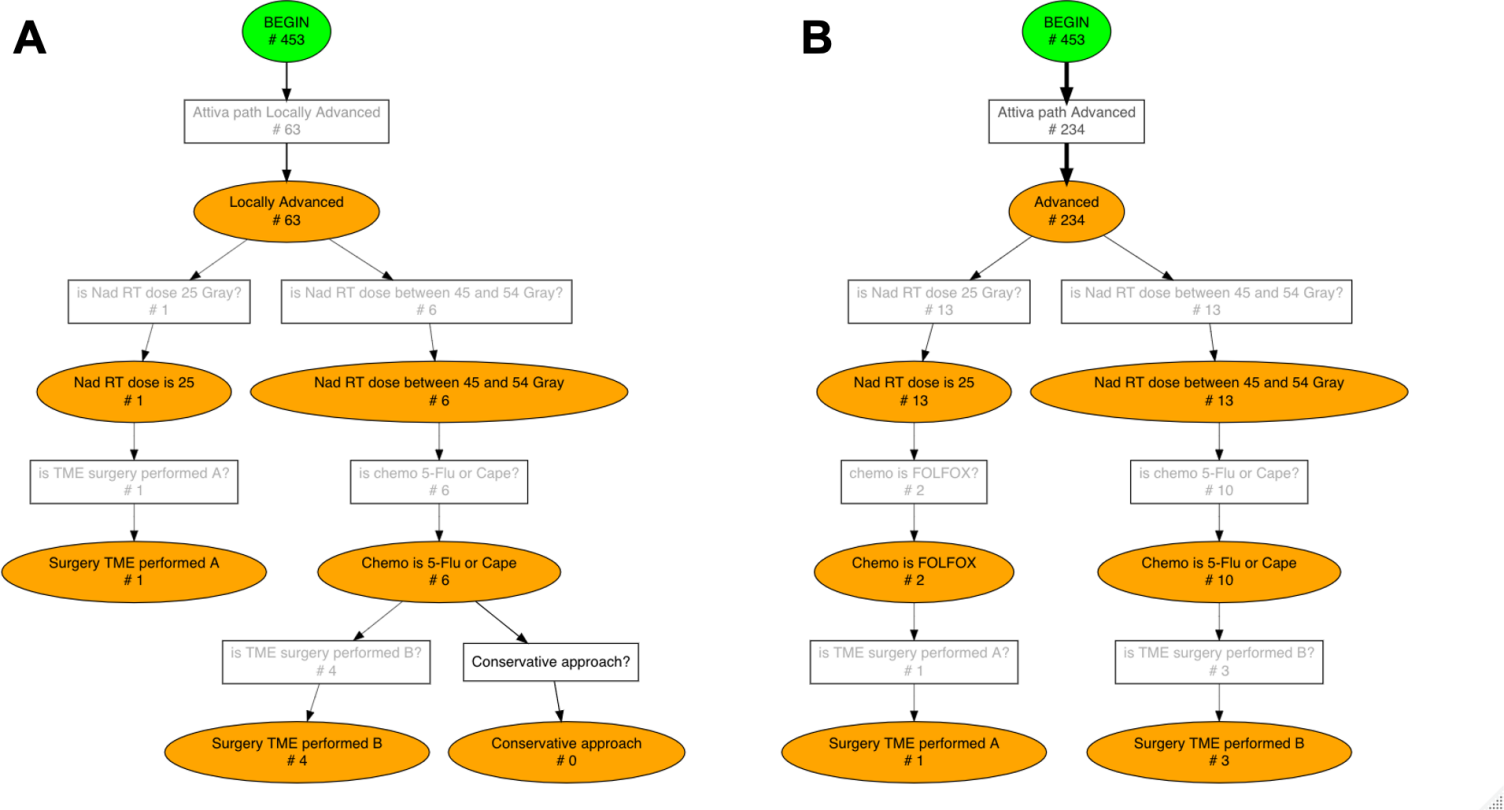
Conformance checking results for Locally Advanced **(A)** and Advanced **(B)** risk groups. The number of patients who activated each rule, i.e. each transition, is displayed on the Pseudo-Workflow diagrams representing ESMO guidelines’ recommendations: boxes are rules, circles are status. Only patients that reach final nodes are patients fully compliant with ESMO guidelines, all others deviate from the guidelines at some point in their treatment pathway.

The PWF diagram corresponding to the ESMO CGs for each risk group is shown in **Figures 5**, **6**. The diagram is composed of boxes representing rules and circles representing statuses, when a rule’s condition is met the engine activates transition to the corresponding status. According to its clinical staging a patient enters one of the four branches of ESMO guidelines, subsequent layers of PWF diagram describe treatment options for each risk category: short course radiotherapy, long course radiotherapy or surgery.

An artificial ‘BEGIN’ node was added to the workflow to represent the starting point of each trace.

The four XML files for PWL guidelines, one for each TNM risk category, are available in the **Supplementary Material**.

### 3.3 Conformance checking analysis

Guidelines adherence was investigated separately for each TNM risk category. The pMineR software automatically computes the number of patients who fulfil each condition of the guidelines represented by the PWF diagram. **Figures 5**, **6** show the results of the conformance checking analysis on the PWF version of the ESMO guidelines.

46 (10.15%) out of 453 patients were in the ‘Early’ TNM category (**Figure 5A**). In this group 11 (23.91%) patients directly underwent TME surgery whereas no patient started the long course treatment with total delivered and fraction doses in the guidelines ranges (total delivered dose between 45 and 54 Gray, fraction dose between 1.8 and 2.0 Gray).

The ‘Intermediate’ risk category involves the wider range of treatment options. In our cohort 110 (24.28%) patients were in the Intermediate category (**Figure 5B**) and they experienced the following treatment pathways: 9 (8.18%) patients entered the short course branch with a radiotherapy dose of 25 Gray and then 6 (5.45%) of them performed surgery TME as required; 10 (9.09%) patients entered the long course branch but only 9 (8.18%) completed it with 5-Flu/Cape chemotherapy followed by TME surgery; 5 (4.54%) patients directly underwent TME surgery.

63 (13.91%) patients of our cohort activated the ‘Locally Advanced’ path (**Figure 6A**). In the “Locally Advanced” group only 1 patient completed the short course treatment including radiotherapy of 25 Gray and TME surgery; 6 (9.52%) patients entered long course path and only 4 (6.35%) concluded it with 5-Flu/Cape chemotherapy followed by TME surgery.

Finally, 234 (51.66%) patients out of 453 were in the ‘Advanced’ category (**Figure 6B**) and they underwent the following treatment pathways: 13 (5.56%) patients entered short course and Folfox chemotherapy was administered to only 2 of them; 13 (5.56%) patients entered long course path and 3 (1.28%) concluded it with 5-Flu/Cape chemotherapy followed by TME surgery.

In each TNM risk group a significant fraction of patient 1) did not enter any guidelines branch or 2) deviate at some point during treatment process.

Distribution of not compliant patients across four risk categories was: 35 (76.09%) in ‘Early’ patients, 90 (81.82%) in ‘Intermediate’ patients, 58 (92.06%) in ‘Locally advanced’ patients, 230 (98.29%) in ‘Advanced’ patients.

**Figure 7:**
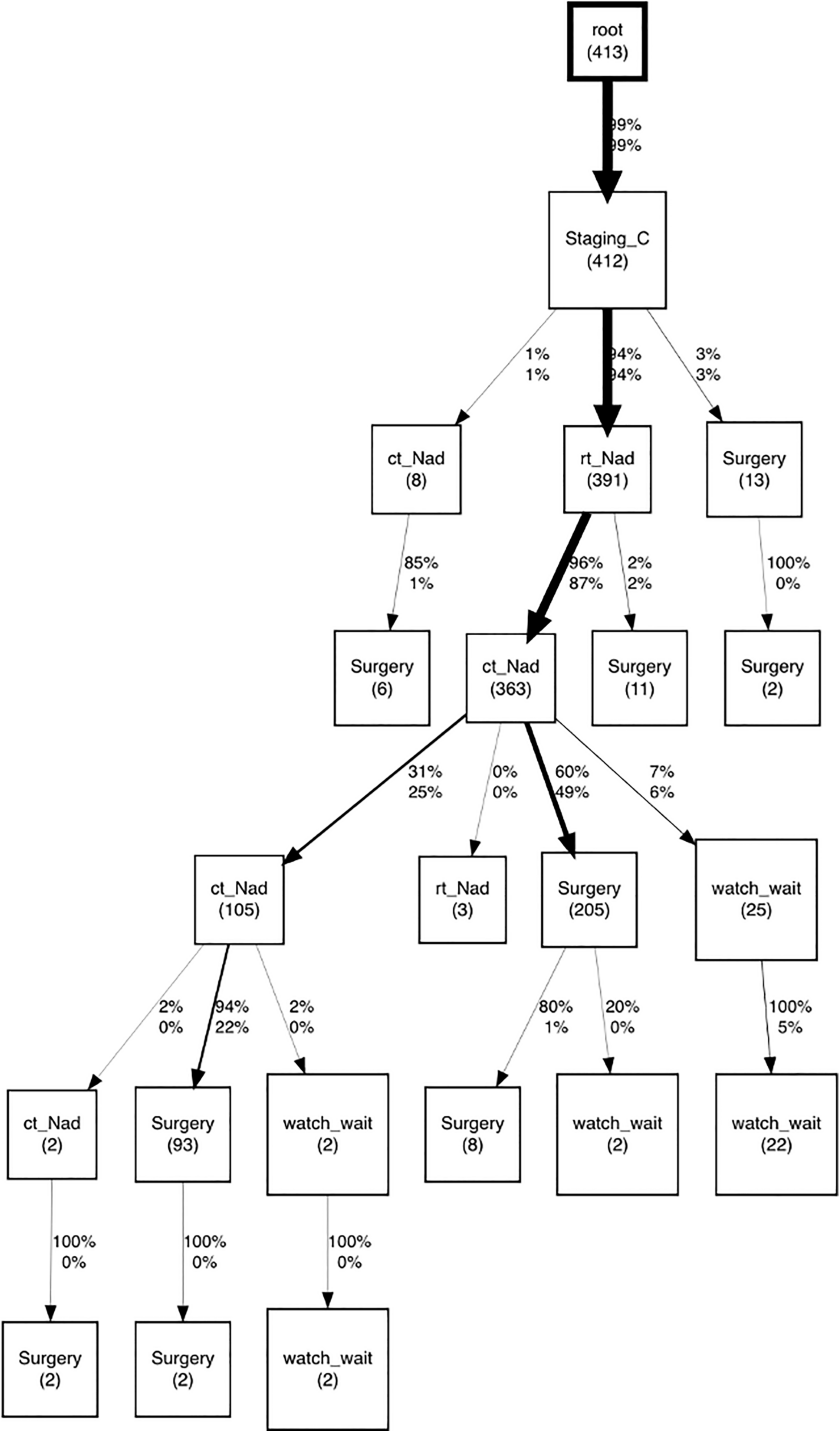
CareFlow miner graph produced with pMineR software to represent the most common processes of patients not compliant to clinical guidelines. Numbers indicate patients passing through each node. The direct acyclic graph displays only transitions involving a number of patients greater than the threshold, set to 4.

About the discovery process with the CareFlow miner algorithm, the most frequent careflow mined was neoadjuvant treatment composed of radiotherapy (rt_Nad) followed by chemotherapy (ct_Nad), as marked with thicker black arrows in CareFlow graph (**Figure 7**).

By inspecting event log of these patients we observed that main reasons of deviation from guidelines recommendations were: 1) a radiotherapy dose slightly above guidelines range for long course treatment 2) Chemotherapy treatment with oxaliplatin, an antineoplastic drug not included in ESMO guidelines.

## 4 Discussion

The research area of CIGs proposes a set of languages to formalize clinical knowledge expressed in natural language within clinical guidelines and make it usable by computer programs (20).

On one side, these languages must be abstract enough to be interpretable by healthcare professionals without a modelling background. On the other side, they must be interpretable by engines that execute guidelines (21).

In this study we employ Pseudo-Workflow formalism, implemented in the pMineR software, to translate clinical guidelines into a computer-interpretable format and to assess adherence in a real-world cohort.

Conformance checking analysis performed with the pMineR software on a real-world cohort of rectal cancer patients allowed us to assess actual conformity to the international guidelines in clinical practice.

A fraction of patients in our cohort received neoadjuvant radiochemotherapy that deviated from the ESMO clinical guidelines, and we observed that the main reasons for these deviations were a moderate increase in radiotherapy dose compared to the current guidelines range or the addition of oxaliplatin to the concurrent chemotherapy schedule. The reasons for this deviation are mainly related to internal protocols that allow dose intensification on the rectal tumor and the corresponding mesorectum up to 55Gy (35). In addition, in patients with high risk factors, such as the presence of positive extramesorectal lymph nodes, tumour deposits, extramural vascular invasion (EMVI), the intensification of concomitant chemotherapy with oxaliplatin in addition to fluoropyrimidine is considered (36).

Today, some of the protocols that deviate from the guidelines are used in our department, as was subsequently reported by publications in the literature in the following years.

The authors believe that the PWF is a suitable formalism for formalizing large and complex guidelines including several treatment options, with results that are easy to interpret in a clinical setting. The basic units, named rules, allow to easily chain the flow of guidelines recommendations as logical conditions and status changes.

Simplicity is one of the key features of the PWF, which is both a limitation and a strength.

A simple structure and an easily interpretable overview of the results, in the form of workflow diagrams are the main advantages of the presented formalism. In a clinical setting, where CIGs need to be validated by a clinician, PWF promotes multidisciplinary teamwork by improving clinician understanding and facilitating collaboration between clinical and technical team.

Furthermore, in the process mining manifesto published by Van Der Aalst W. et al. in 2012 (11), simplicity is indicated as one of the four quality measures for a process model, to be balanced with generalizability, precision and fit.

However, the simple structure of the proposed formalism provides a limited set of constructs. One of the main limitations is the lack of a construct that evaluates how long a rule has been active, e.g. to define a threshold for the time interval between the end of the treatment and the surgery. However, this limitation can be addressed during the pre-processing phase by creating some *ad hoc* attributes that indicate the time interval from a given event.

In addition, for future work, the implementation of both hard and soft constraints in PWF would allow to define a degree of conformance or deviation instead of a binary response “adherent vs. nonadherent”. This would increase the interpretability and flexibility of the conformance algorithm and detect situations of small deviations from clinical guidelines.

We believe that the data mining strategy emerging from the PWF could form the basis for further clinical analysis that will be aimed at gaining a deeper understanding of the applicability of the protocols used to date. Moreover, further analyses are needed to assess the impact of adherence and nonadherence to CGs on oncological outcomes such as overall survival, disease free survival, metastases free survival, and local control.

## 5 Conclusions

In this study we assessed conformity to international ESMO guidelines in a real-world cohort of rectal cancer patients. ESMO guidelines were translated into a computer-interpretable version using the Pseudo-Workflow formalism and pMineR software was employed to perform conformance checking analysis.

The Pseudo-Workflow formalism proved to be a powerful method for representing standard international guidelines for rectal cancer treatment in a computer-interpretable format and for checking adherence using process mining techniques. PWF is characterized by a simple structure, and simplicity is also one of its strengths, allowing clinical guidelines to be represented in an easy and interpretable manner, enhancing multidisciplinary collaboration between clinician and technical team.

Real-world application to a cohort of rectal cancer patients identified a subgroup of patients who did not adhere to clinical guidelines during their treatment process due to hospital internal protocols.

Process mining techniques provided healthcare professionals with useful tools to investigate actual guidelines adherence, further analysis will assess the impact of clinical guidelines compliance on significant clinical outcomes.

## Data availability statement

The datasets presented in this article are not readily available because of privacy/ethical restrictions. Aggregated data generated for this study are available on request. Requests to access the datasets should be directed to mariachiara.savino@unicatt.it.

## Ethics statement

The studies involving human participants were reviewed and approved by Ethics committee, Fondazione Policlinico Gemelli IRCCS, Rome. Written informed consent for participation was not required for this study in accordance with the national legislation and the institutional requirements.

## Author contributions

MS, GC, CM, JL, MG, VV, AD: concept. MS, GC: analysis execution. MS: data extraction and pre-processing. NC, BG, JL: draft revision. All authors have contributed to the design and assessment of results of the study as well as read and approved the manuscript. All authors contributed to the article and approved the submitted version.

## Footnotes

1 Available on GitHub at http://github.com/PMLiquidLab
