# Supplementary material for: A process mining approach for clinical guidelines compliance: real-world application in rectal cancer
Source: Front Oncol. 2023 May 17;13:1090076. doi: 10.3389/fonc.2023.1090076 (PMC10231435; doi:10.3389/fonc.2023.1090076)
Supplement: Supplementary file 1 [file DataSheet_1.pdf]

Supplementary material

ESMO guidelines in Pseudo-Workflow formalism

1.1 Early

```
<xml>
  <workflow>
    <node name='BEGIN'></node>
    <node name='Early'></node>
    <node name='Nad RT dose between 45 and 54 Gray'></node>
    <node name='Chemo is 5-Flu or Cape'></node>
    <node name='Local excision performed'></node>
    <node name='Watch and wait'></node>
    <node name='Surgery TME performed'></node>
    <node name='Nad RT fuori linea guida B' plotIt = 'FALSE'>
    </node>


 <trigger name='Attiva path Early'>
        <condition>'BEGIN' %in% $st.ACTIVE$ AND $ev.NOW$=='Staging_C' AND
$ev.NOW::attr('type_disease')$== 'Early'  </condition>
        <set>'Early'</set>
        <unset>'BEGIN'</unset>
    </trigger>


<trigger  name='is Nad RT dose between 45 and 54 Gray?'>
      <condition>'Early' %in% $st.ACTIVE$ AND $ev.NOW$=='rt_Nad' AND
as.numeric($ev.NOW::attr('TotalDoseT')$)&gt;= as.numeric('45.0') AND
as.numeric($ev.NOW::attr('TotalDoseT')$)&lt;= as.numeric('54.0') AND
as.numeric($ev.NOW::attr('FractionDoseT')$)&gt;= as.numeric('1.8') AND
as.numeric($ev.NOW::attr('FractionDoseT')$)&lt;= as.numeric('2.0')</condition>
      <set>'Nad RT dose between 45 and 54 Gray'</set>
      <unset>'Early'</unset>
    </trigger>


    <trigger  name='is chemo 5-Flu or Cape?'>
      <condition>'Nad RT dose between 45 and 54 Gray' %in% $st.ACTIVE$ AND
$ev.NOW$== 'ct_Nad' AND $ev.NOW::attr('Ox')$!= '1' AND
($ev.NOW::attr('farmaci_lineaguida')$== 'Capecitabine' OR
$ev.NOW::attr('farmaci_lineaguida')$== '5-Flouroulacil')</condition>
      <set>'Chemo is 5-Flu or Cape'</set>
      <unset>'Nad RT dose between 45 and 54 Gray'</unset>
     </trigger>


    <trigger  name='Nad RT e CT fuori linea guida B?' plotIt = 'FALSE'>
```

```
      <condition>'Early' %in% $st.ACTIVE$ AND (($ev.NOW$== 'rt_Nad' AND
(as.numeric($ev.NOW::attr('TotalDoseT')$)&lt; as.numeric('45.0') OR
as.numeric($ev.NOW::attr('TotalDoseT')$)&gt; as.numeric('54.0') OR
as.numeric($ev.NOW::attr('FractionDoseT')$)&lt; as.numeric('1.8') OR
as.numeric($ev.NOW::attr('FractionDoseT')$)&gt; as.numeric('2.0'))) OR
($ev.NOW$== 'ct_Nad' AND $ev.NOW::attr('Ox')$== '1') OR ($ev.NOW$== 'ct_Nad' AND
$ev.NOW::attr('farmaci_lineaguida')$!= 'Capecitabine' AND
$ev.NOW::attr('farmaci_lineaguida')$!= '5-Flouroulacil' ) OR
($ev.NOW::attr('SurgeryTechnique')$== 'TEM' OR
$ev.NOW::attr('SurgeryTechnique')$== 'Transanal excision' OR
$ev.NOW::attr('TypeLocalSurgery')$== 'Local excision')) </condition>
      <set>'Nad RT fuori linea guida B'</set>
      <unset>'Early'</unset>
    </trigger>


    <trigger  name='is Local Excision performed?'>
      <condition>'Chemo is 5-Flu or Cape' %in% $st.ACTIVE$ AND
$ev.NOW$=='Surgery' AND $ev.NOW::attr('TypeLocalSurgery')$== 'Local excision'
</condition>
      <set>'Local excision performed'</set>
      <unset>'Chemo is 5-Flu or Cape'</unset>
    </trigger>


    <trigger  name='Watch and wait B?'>
      <condition>'Chemo is 5-Flu or Cape' %in% $st.ACTIVE$ AND
$ev.NOW$=='watch_wait'</condition>
      <set>'Watch and wait'</set>
      <unset>'Chemo is 5-Flu or Cape'</unset>
    </trigger>


    <trigger  name='is TME surgery performed?'>
      <condition>'Early' %in% $st.ACTIVE$ AND !('Nad RT fuori linea guida B' %in%
$st.ACTIVE$) AND $ev.NOW$=='Surgery' AND ($ev.NOW::attr('SurgeryTechnique')$==
'TME' OR $ev.NOW::attr('SurgeryTechnique')$== 'TaTME' OR
$ev.NOW::attr('SurgeryTechnique')$== 'PME' OR
$ev.NOW::attr('TypeLocalSurgery')$== 'APR' OR
$ev.NOW::attr('TypeLocalSurgery')$== 'Anterior resection' OR
$ev.NOW::attr('TypeLocalSurgery')$== 'LAR') </condition>
      <set>'Surgery TME performed'</set>
      <unset>'Early'</unset>
    </trigger>

  </workflow>
</xml>
```

1.2 Intermediate

```
<xml>
  <workflow>

   <node name='Intermediate'></node>
    <node name='Nad RT dose is 25'></node>
    <node name='Nad RT e CT fuori linea guida short C' plotIt = 'FALSE'></node>
    <node name='Surgery TME performed A'></node>
    <node name='Nad RT dose between 45 and 54 Gray'></node>
    <node name='Chemo is 5-Flu or Cape'></node>
    <node name='Conservative approach'></node>
    <node name='Surgery TME performed B'></node>
    <node name='Nad RT e CT fuori linea guida' plotIt = 'FALSE'></node>
    <node name='Surgery TME performed C'></node>



   <trigger name='Attiva path Intermediate'>
        <condition>'BEGIN' %in% $st.ACTIVE$ AND $ev.NOW$=='Staging_C' AND
$ev.NOW::attr('type_disease')$== 'Intermediate'</condition>
        <set>'Intermediate'</set>
        <unset>'BEGIN'</unset>
    </trigger>

    <trigger name='is Nad RT dose 25 Gray?'>
        <condition>'Intermediate' %in% $st.ACTIVE$ AND $ev.NOW$=='rt_Nad' AND
as.numeric($ev.NOW::attr('TotalDoseT')$)== as.numeric('25.0') AND
as.numeric($ev.NOW::attr('FractionDoseT')$)== as.numeric('5.0')</condition>
        <set>'Nad RT dose is 25'</set>
        <unset>'Intermediate'</unset>
    </trigger>


    <trigger  name='Nad RT e CT fuori linea guida short C?' plotIt = 'FALSE'>
      <condition>'Nad RT dose is 25 C' %in% $st.ACTIVE$ AND ($ev.NOW$=='ct_Nad'
OR $ev.NOW$=='rt_Nad' OR $ev.NOW::attr('SurgeryTechnique')$== 'TEM' OR
$ev.NOW::attr('SurgeryTechnique')$== 'Transanal excision' OR
$ev.NOW::attr('TypeLocalSurgery')$== 'Local excision') </condition>
      <set>'Nad RT e CT fuori linea guida short C'</set>
      <unset>'Nad RT dose is 25 C'</unset>
    </trigger>

    <trigger name='is TME surgery performed A?'>
        <condition>'Nad RT dose is 25' %in% $st.ACTIVE$ AND !('Nad RT e CT fuori
linea guida short C' %in% $st.ACTIVE$) AND $ev.NOW$=='Surgery' AND
($ev.NOW::attr('SurgeryTechnique')$== 'TME' OR
$ev.NOW::attr('SurgeryTechnique')$== 'TaTME' OR
$ev.NOW::attr('SurgeryTechnique')$== 'PME' OR
$ev.NOW::attr('TypeLocalSurgery')$== 'APR' OR
$ev.NOW::attr('TypeLocalSurgery')$== 'Anterior resection' OR
$ev.NOW::attr('TypeLocalSurgery')$== 'LAR')</condition>
        <set>'Surgery TME performed A'</set>
        <unset>'Nad RT dose is 25'</unset>
    </trigger>

    <trigger  name='is Nad RT dose between 45 and 54 Gray?'>
```

```
      <condition>'Intermediate' %in% $st.ACTIVE$ AND $ev.NOW$=='rt_Nad' AND
as.numeric($ev.NOW::attr('TotalDoseT')$)&gt;= as.numeric('45.0') AND
as.numeric($ev.NOW::attr('TotalDoseT')$)&lt;= as.numeric('54.0') AND
as.numeric($ev.NOW::attr('FractionDoseT')$)&gt;= as.numeric('1.8') AND
as.numeric($ev.NOW::attr('FractionDoseT')$)&lt;= as.numeric('2.0') </condition>
      <set>'Nad RT dose between 45 and 54 Gray'</set>
      <unset>'Intermediate'</unset>
    </trigger>


    <trigger  name='is chemo 5-Flu or Cape?'>
      <condition>'Nad RT dose between 45 and 54 Gray' %in% $st.ACTIVE$ AND
$ev.NOW$== 'ct_Nad' AND $ev.NOW::attr('Ox')$!= '1' AND
($ev.NOW::attr('farmaci_lineaguida')$== 'Capecitabine' OR
$ev.NOW::attr('farmaci_lineaguida')$== '5-Flouroulacil')</condition>
      <set>'Chemo is 5-Flu or Cape'</set>
      <unset>'Nad RT dose between 45 and 54 Gray'</unset>
    </trigger>


    <trigger name='is TME surgery performed B?'>
        <condition>'Chemo is 5-Flu or Cape' %in% $st.ACTIVE$ AND
$ev.NOW$=='Surgery' AND ($ev.NOW::attr('SurgeryTechnique')$== 'TME' OR
$ev.NOW::attr('SurgeryTechnique')$== 'TaTME' OR
$ev.NOW::attr('SurgeryTechnique')$== 'PME' OR
$ev.NOW::attr('TypeLocalSurgery')$== 'APR' OR
$ev.NOW::attr('TypeLocalSurgery')$== 'Anterior resection' OR
$ev.NOW::attr('TypeLocalSurgery')$== 'LAR')</condition>
        <set>'Surgery TME performed B'</set>
        <unset>'Chemo is 5-Flu or Cape'</unset>
    </trigger>


    <trigger name='Conservative approach?'>
        <condition>'Chemo is 5-Flu or Cape' %in% $st.ACTIVE$ AND
($ev.NOW$=='watch_wait' OR $ev.NOW::attr('SurgeryTechnique')$== 'TEM' OR
$ev.NOW::attr('SurgeryTechnique')$== 'Transanal excision' OR
$ev.NOW::attr('TypeLocalSurgery')$== 'Local excision')</condition>
        <set>'Conservative approach'</set>
        <unset>'Chemo is 5-Flu or Cape'</unset>
    </trigger>


    <trigger  name='Nad RT e CT fuori linea guida E?' plotIt = 'FALSE'>
      <condition>'Intermediate' %in% $st.ACTIVE$ AND (($ev.NOW$=='rt_Nad' AND
((as.numeric($ev.NOW::attr('TotalDoseT')$)&lt; as.numeric('45.0') AND
as.numeric($ev.NOW::attr('TotalDoseT')$)!=as.numeric('25.0')) OR
(as.numeric($ev.NOW::attr('TotalDoseT')$)&gt; as.numeric('54.0')) OR
(as.numeric($ev.NOW::attr('FractionDoseT')$)&lt; as.numeric('1.8')) OR
(as.numeric($ev.NOW::attr('FractionDoseT')$)&gt; as.numeric('2.0') AND
as.numeric($ev.NOW::attr('FractionDoseT')$)!= as.numeric('5.0')))) OR ($ev.NOW$==
'ct_Nad' AND $ev.NOW::attr('Ox')$== '1') OR ($ev.NOW$== 'ct_Nad' AND
$ev.NOW::attr('farmaci_lineaguida')$!= 'Capecitabine' AND
$ev.NOW::attr('farmaci_lineaguida')$!= '5-Flouroulacil')  OR
($ev.NOW::attr('SurgeryTechnique')$== 'TEM' OR
$ev.NOW::attr('SurgeryTechnique')$== 'Transanal excision' OR
$ev.NOW::attr('TypeLocalSurgery')$== 'Local excision')) </condition>
```

```
      <set>'Nad RT e CT fuori linea guida'</set>
      <unset>'Intermediate'</unset>
    </trigger>


    <trigger name='is TME surgery performed C?'>
        <condition>'Intermediate' %in% $st.ACTIVE$ AND !('Nad RT fuori linea
guida' %in% $st.ACTIVE$) AND $ev.NOW$=='Surgery' AND
($ev.NOW::attr('SurgeryTechnique')$== 'TME' OR
$ev.NOW::attr('SurgeryTechnique')$== 'TaTME' OR
$ev.NOW::attr('SurgeryTechnique')$== 'PME' OR
$ev.NOW::attr('TypeLocalSurgery')$== 'APR' OR
$ev.NOW::attr('TypeLocalSurgery')$== 'Anterior resection' OR
$ev.NOW::attr('TypeLocalSurgery')$== 'LAR') </condition>
        <set>'Surgery TME performed C'</set>
        <unset>'Intermediate'</unset>
    </trigger>


    </workflow>
</xml>
```

1.3 Locally Advanced

```
<xml>
  <workflow>
  <node name='Locally Advanced'></node>
    <node name='Nad RT dose is 25'></node>
    <node name='Nad RT e CT fuori linea guida short F' plotIt = 'FALSE'></node>
    <node name='Surgery TME performed A'></node>
    <node name='Nad RT dose between 45 and 54 Gray'></node>
    <node name='Chemo is 5-Flu or Cape'></node>
    <node name='Surgery TME performed B'></node>
    <node name='Conservative approach'></node>
    <node name='Nad RT e CT fuori linea guida G' plotIt = 'FALSE'></node>



    <trigger name='Attiva path Locally Advanced'>
        <condition>'BEGIN' %in% $st.ACTIVE$ AND $ev.NOW$=='Staging_C' AND
$ev.NOW::attr('type_disease')$== 'Locally advanced'</condition>
        <set>'Locally Advanced'</set>
        <unset>'BEGIN'</unset>
    </trigger>

    <trigger name='is Nad RT dose 25 Gray?'>
        <condition>'Locally Advanced' %in% $st.ACTIVE$ AND $ev.NOW$=='rt_Nad' AND
as.numeric($ev.NOW::attr('TotalDoseT')$)== as.numeric('25.0') AND
as.numeric($ev.NOW::attr('FractionDoseT')$)== as.numeric('5.0')</condition>
        <set>'Nad RT dose is 25'</set>
        <unset>'Locally Advanced'</unset>
    </trigger>

    <trigger  name='Nad RT e CT fuori linea guida short F?' plotIt = 'FALSE'>
      <condition>'Nad RT dose is 25' %in% $st.ACTIVE$ AND ($ev.NOW$=='ct_Nad' OR
$ev.NOW$=='rt_Nad' OR $ev.NOW::attr('SurgeryTechnique')$== 'TEM' OR
$ev.NOW::attr('SurgeryTechnique')$== 'Transanal excision' OR
$ev.NOW::attr('TypeLocalSurgery')$== 'Local excision') </condition>
      <set>'Nad RT e CT fuori linea guida short F'</set>
      <unset>'Nad RT dose is 25'</unset>
    </trigger>

    <trigger name='is TME surgery performed A?'>
        <condition>'Nad RT dose is 25' %in% $st.ACTIVE$ AND !('Nad RT e CT fuori
linea guida short F' %in% $st.ACTIVE$) AND $ev.NOW$=='Surgery' AND
($ev.NOW::attr('SurgeryTechnique')$== 'TME' OR
$ev.NOW::attr('SurgeryTechnique')$== 'TaTME' OR
$ev.NOW::attr('SurgeryTechnique')$== 'PME' OR
$ev.NOW::attr('TypeLocalSurgery')$== 'APR' OR
$ev.NOW::attr('TypeLocalSurgery')$== 'Anterior resection' OR
$ev.NOW::attr('TypeLocalSurgery')$== 'LAR') </condition>
        <set>'Surgery TME performed A'</set>
        <unset>'Nad RT dose is 25'</unset>
    </trigger>

    <trigger  name='is Nad RT dose between 45 and 54 Gray?'>
```

```
      <condition>'Locally Advanced' %in% $st.ACTIVE$ AND $ev.NOW$=='rt_Nad' AND
as.numeric($ev.NOW::attr('TotalDoseT')$)&gt;= as.numeric('45.0') AND
as.numeric($ev.NOW::attr('TotalDoseT')$)&lt;= as.numeric('54.0') AND
as.numeric($ev.NOW::attr('FractionDoseT')$)&gt;= as.numeric('1.8') AND
as.numeric($ev.NOW::attr('FractionDoseT')$)&lt;= as.numeric('2.0')</condition>
      <set>'Nad RT dose between 45 and 54 Gray'</set>
      <unset>'Locally Advanced'</unset>
    </trigger>

    <trigger  name='is chemo 5-Flu or Cape?'>
      <condition>'Nad RT dose between 45 and 54 Gray' %in% $st.ACTIVE$ AND
$ev.NOW$== 'ct_Nad' AND $ev.NOW::attr('Ox')$!= '1' AND
($ev.NOW::attr('farmaci_lineaguida')$== 'Capecitabine' OR
$ev.NOW::attr('farmaci_lineaguida')$== '5-Flouroulacil')</condition>
      <set>'Chemo is 5-Flu or Cape'</set>
      <unset>'Nad RT dose between 45 and 54 Gray'</unset>
    </trigger>

    <trigger name='is TME surgery performed B?'>
        <condition>'Chemo is 5-Flu or Cape' %in% $st.ACTIVE$ AND
$ev.NOW$=='Surgery' AND ($ev.NOW::attr('SurgeryTechnique')$== 'TME' OR
$ev.NOW::attr('SurgeryTechnique')$== 'TaTME' OR
$ev.NOW::attr('SurgeryTechnique')$== 'PME' OR
$ev.NOW::attr('TypeLocalSurgery')$== 'APR' OR
$ev.NOW::attr('TypeLocalSurgery')$== 'Anterior resection' OR
$ev.NOW::attr('TypeLocalSurgery')$== 'LAR') </condition>
        <set>'Surgery TME performed B'</set>
        <unset>'Chemo is 5-Flu or Cape'</unset>
    </trigger>

    <trigger name='Conservative approach?'>
        <condition>'Chemo is 5-Flu or Cape' %in% $st.ACTIVE$ AND
($ev.NOW$=='watch_wait' OR $ev.NOW::attr('SurgeryTechnique')$== 'TEM' OR
$ev.NOW::attr('SurgeryTechnique')$== 'Transanal excision' OR
$ev.NOW::attr('TypeLocalSurgery')$== 'Local excision')</condition>
        <set>'Conservative approach'</set>
        <unset>'Chemo is 5-Flu or Cape'</unset>
    </trigger>

   <trigger  name='Nad RT e CT fuori linea guida G?' plotIt = 'FALSE'>
      <condition>'Locally Advanced' %in% $st.ACTIVE$ AND (($ev.NOW$=='rt_Nad' AND
((as.numeric($ev.NOW::attr('TotalDoseT')$)&lt; as.numeric('45.0') AND
as.numeric($ev.NOW::attr('TotalDoseT')$)!=as.numeric('25.0')) OR
(as.numeric($ev.NOW::attr('TotalDoseT')$)&gt; as.numeric('54.0')) OR
(as.numeric($ev.NOW::attr('FractionDoseT')$)&lt; as.numeric('1.8')) OR
(as.numeric($ev.NOW::attr('FractionDoseT')$)&gt; as.numeric('2.0') AND
as.numeric($ev.NOW::attr('FractionDoseT')$)!= as.numeric('5.0')))) OR ($ev.NOW$==
'ct_Nad' AND $ev.NOW::attr('Ox')$== '1') OR ($ev.NOW$== 'ct_Nad' AND
$ev.NOW::attr('farmaci_lineaguida')$!= 'Capecitabine' AND
$ev.NOW::attr('farmaci_lineaguida')$!= '5-Flouroulacil')  OR
($ev.NOW::attr('SurgeryTechnique')$== 'TEM' OR
$ev.NOW::attr('SurgeryTechnique')$== 'Transanal excision' OR
$ev.NOW::attr('TypeLocalSurgery')$== 'Local excision')) </condition>
      <set>'Nad RT e CT fuori linea guida G'</set>
      <unset>'Locally Advanced'</unset>
    </trigger>

    </workflow>
</xml>
```

1.4 Advanced

```
<xml>
  <workflow>

    <node name='Advanced'></node>
    <node name='Nad RT dose is 25'></node>
    <node name='Chemo is FOLFOX'></node>
    <node name='Surgery TME performed A'></node>
    <node name='Nad RT dose between 45 and 54 Gray'></node>
    <node name='Chemo is 5-Flu or Cape'></node>
    <node name='Surgery TME performed B'></node>
    <node name='Nad RT e CT fuori linea guida I' plotIt = 'FALSE'></node>
    <node name='Fuori linee guida short' plotIt = 'FALSE'></node>
    <node name='Fuori linee guida long' plotIt = 'FALSE'></node>


    <trigger name='Attiva path Advanced'>
        <condition>'BEGIN' %in% $st.ACTIVE$ AND $ev.NOW$=='Staging_C' AND
$ev.NOW::attr('type_disease')$== 'Advanced'</condition>
        <set>'Advanced'</set>
        <unset>'BEGIN'</unset>
    </trigger>

    <trigger name='is Nad RT dose 25 Gray?'>
        <condition>'Advanced' %in% $st.ACTIVE$ AND $ev.NOW$=='rt_Nad' AND
as.numeric($ev.NOW::attr('TotalDoseT')$)== as.numeric('25.0') AND
as.numeric($ev.NOW::attr('FractionDoseT')$)== as.numeric('5.0')</condition>
        <set>'Nad RT dose is 25'</set>
        <unset>'Advanced'</unset>
    </trigger>

    <trigger  name='chemo is FOLFOX?'>
      <condition>'Nad RT dose is 25' %in% $st.ACTIVE$ AND $ev.NOW$== 'ct_Nad' AND
$ev.NOW::attr('folfox')$== '1' </condition>
      <set>'Chemo is FOLFOX'</set>
      <unset>'Nad RT dose is 25'</unset>
    </trigger>

    <trigger name='is TME surgery performed A?'>
        <condition>'Chemo is FOLFOX' %in% $st.ACTIVE$ AND  !('Fuori linee guida
short' %in% $st.ACTIVE$) AND $ev.NOW$=='Surgery' AND
($ev.NOW::attr('SurgeryTechnique')$== 'TME' OR
$ev.NOW::attr('SurgeryTechnique')$== 'TaTME' OR
$ev.NOW::attr('SurgeryTechnique')$== 'PME' OR
$ev.NOW::attr('TypeLocalSurgery')$== 'APR' OR
$ev.NOW::attr('TypeLocalSurgery')$== 'Anterior resection' OR
$ev.NOW::attr('TypeLocalSurgery')$== 'LAR') </condition>
        <set>'Surgery TME performed A'</set>
        <unset>'Chemo is FOLFOX'</unset>
    </trigger>

    <trigger  name='is Nad RT dose between 45 and 54 Gray?'>
      <condition>'Advanced' %in% $st.ACTIVE$ AND $ev.NOW$=='rt_Nad' AND
as.numeric($ev.NOW::attr('TotalDoseT')$)&gt;= as.numeric('45.0') AND
```

```
as.numeric($ev.NOW::attr('TotalDoseT')$)&lt;= as.numeric('54.0') AND
as.numeric($ev.NOW::attr('FractionDoseT')$)&gt;= as.numeric('1.8') AND
as.numeric($ev.NOW::attr('FractionDoseT')$)&lt;= as.numeric('2.0')</condition>
      <set>'Nad RT dose between 45 and 54 Gray'</set>
      <unset>'Advanced'</unset>
    </trigger>

    <trigger  name='is chemo 5-Flu or Cape?'>
      <condition>'Nad RT dose between 45 and 54 Gray' %in% $st.ACTIVE$ AND
$ev.NOW$== 'ct_Nad' AND $ev.NOW::attr('Ox')$!= '1' AND
($ev.NOW::attr('farmaci_lineaguida')$== 'Capecitabine' OR
$ev.NOW::attr('farmaci_lineaguida')$== '5-Flouroulacil')</condition>
      <set>'Chemo is 5-Flu or Cape'</set>
      <unset>'Nad RT dose between 45 and 54 Gray'</unset>
    </trigger>

    <trigger name='is TME surgery performed B?'>
        <condition>'Chemo is 5-Flu or Cape' %in% $st.ACTIVE$ AND !('Fuori linee
guida long' %in% $st.ACTIVE$) AND $ev.NOW$=='Surgery' AND
($ev.NOW::attr('SurgeryTechnique')$== 'TME' OR
$ev.NOW::attr('SurgeryTechnique')$== 'TaTME' OR
$ev.NOW::attr('SurgeryTechnique')$== 'PME' OR
$ev.NOW::attr('TypeLocalSurgery')$== 'APR' OR
$ev.NOW::attr('TypeLocalSurgery')$== 'Anterior resection' OR
$ev.NOW::attr('TypeLocalSurgery')$== 'LAR') </condition>
        <set>'Surgery TME performed B'</set>
        <unset>'Chemo is 5-Flu or Cape'</unset>
    </trigger>

     <trigger  name='Nad RT e CT fuori linea guida I?' plotIt = 'FALSE'>
      <condition>'Advanced' %in% $st.ACTIVE$ AND (($ev.NOW$=='rt_Nad' AND
((as.numeric($ev.NOW::attr('TotalDoseT')$)&lt; as.numeric('45.0') AND
as.numeric($ev.NOW::attr('TotalDoseT')$)!=as.numeric('25.0')) OR
(as.numeric($ev.NOW::attr('TotalDoseT')$)&gt; as.numeric('54.0')) OR
(as.numeric($ev.NOW::attr('FractionDoseT')$)&lt; as.numeric('1.8')) OR
(as.numeric($ev.NOW::attr('FractionDoseT')$)&gt; as.numeric('2.0') AND
as.numeric($ev.NOW::attr('FractionDoseT')$)!= as.numeric('5.0')))) OR ($ev.NOW$==
'ct_Nad' AND $ev.NOW::attr('Ox')$== '1') OR ($ev.NOW$== 'ct_Nad' AND
$ev.NOW::attr('farmaci_lineaguida')$!= 'Capecitabine' AND
$ev.NOW::attr('farmaci_lineaguida')$!= '5-Flouroulacil')  OR
($ev.NOW::attr('SurgeryTechnique')$== 'TEM' OR
$ev.NOW::attr('SurgeryTechnique')$== 'Transanal excision' OR
$ev.NOW::attr('TypeLocalSurgery')$== 'Local excision')) </condition>
      <set>'Nad RT e CT fuori linea guida I'</set>
      <unset>'Advanced'</unset>
    </trigger>

    <trigger  name='Nad RT e CT fuori linea guida short?' plotIt = 'FALSE'>
      <condition>'Chemo is FOLFOX' %in% $st.ACTIVE$ AND ($ev.NOW$=='ct_Nad' OR
$ev.NOW$=='rt_Nad' OR $ev.NOW::attr('SurgeryTechnique')$== 'TEM' OR
$ev.NOW::attr('SurgeryTechnique')$== 'Transanal excision' OR
$ev.NOW::attr('TypeLocalSurgery')$== 'Local excision') </condition>
      <set>'Fuori linee guida short'</set>
      <unset>'Chemo is FOLFOX'</unset>
    </trigger>

    <trigger  name='Nad RT e CT fuori linea guida long?' plotIt = 'FALSE'>
      <condition>'Chemo is 5-Flu or Cape' %in% $st.ACTIVE$ AND
($ev.NOW$=='ct_Nad' OR $ev.NOW$=='rt_Nad' OR $ev.NOW::attr('SurgeryTechnique')$==
```

```
'TEM' OR $ev.NOW::attr('SurgeryTechnique')$== 'Transanal excision' OR
$ev.NOW::attr('TypeLocalSurgery')$== 'Local excision') </condition>
      <set>'Fuori linee guida long'</set>
      <unset>'Chemo is 5-Flu or Cape'</unset>
    </trigger>

  </workflow>
</xml>
```
